# Fatal Elizabethkingia anophelis Bacteremia Complicated by Meningitis in a Hemodialysis Patient: A Case Report and Review of the Literature

**DOI:** 10.7759/cureus.110853

**Published:** 2026-06-14

**Authors:** Yoshikuni Nagayama, Mariko Hashimoto, Ayana Ichikura-Iida, Takashi Inoue, Hiroyuki Hayashi

**Affiliations:** 1 Nephrology, Yokohama Municipal Citizen's Hospital, Yokohama, JPN; 2 Pathology, Yokohama Municipal Citizen's Hospital, Yokohama, JPN

**Keywords:** bacteremia, dialysis, elizabethkingia, elizabethkingia anophelis, end-stage renal disease, meningitis, nonocclusive mesenteric ischemia

## Abstract

*Elizabethkingia anophelis* (*E. anophelis*​​​​​​) is an emerging multidrug-resistant pathogen in hospital aquatic environments associated with severe infections. Reports of *E. anophelis* infection in hemodialysis patients are rare, and meningitis caused by this organism has been poorly characterized. We report a fatal case of meningitis in a hemodialysis patient caused by *E. anophelis* bacteremia and review previous cases of *E. anophelis* infection in hemodialysis patients. A 68-year-old man with end-stage renal disease due to diabetic kidney disease on maintenance hemodialysis was admitted with fever after recent glucocorticoid therapy. Blood cultures grew *E. anophelis*. Despite initial antimicrobial treatment, the patient developed bacterial meningitis with impaired consciousness and abnormal cerebrospinal fluid (CSF) findings. Although CSF cultures were negative, repeated blood cultures remained positive for *E. anophelis*, and alternative pathogens were excluded using a multiplex meningitis/encephalitis panel. Therefore, meningitis due to *E. anophelis* was considered. Antimicrobial therapy was changed to levofloxacin (LVFX) plus sulfamethoxazole-trimethoprim according to susceptibility testing and CSF penetration, resulting in improvement of inflammatory markers, CSF findings, and consciousness. However, the patient subsequently developed nonocclusive mesenteric ischemia (NOMI) and massive iliopsoas hematomas and died of hemorrhagic shock. Autopsy confirmed NOMI without bowel necrosis, and the cause of death was attributed to hemorrhagic shock with NOMI, not directly related to *E. anophelis* infection. A literature review identified only three cases of *E. anophelis* infection in hemodialysis patients. Unlike our case, those patients had catheter-related bloodstream infection or pneumonia and survived following antimicrobial therapy, most commonly including LVFX. This case highlights that *E. anophelis* can cause meningitis in hemodialysis patients. Given its multidrug resistance and persistence in healthcare aquatic environments, clinicians and dialysis healthcare workers should be aware of this uncommon but potentially life-threatening pathogen.

## Introduction

*Elizabethkingia* spp. comprise aerobic, nonfermenting, Gram-negative bacilli that were previously named *Flavobacterium* or *Chryseobacterium* [1]. The name* Elizabethkingia* is derived from the fact that *Flavobacterium meningosepticum* was first identified in 1959 by Elizabeth O. King as a novel cause of bacterial meningitis in pediatric patients [2]. *Elizabethkingia* spp. are ubiquitous in aquatic environments, such as rivers and soil, and in healthcare settings like plumbing and drains [3]. Although they usually do not become human pathogens, they can cause serious conditions like meningitis and sepsis, with high mortality rates, especially in neonates and immunocompromised patients [4]. The clinically relevant species are *Elizabethkingia anophelis (E. anophelis*), *Elizabethkingia* ​​​​​​*meningoseptica (E. meningoseptica*), and *Elizabethkingia miricola (E. miricola*). Among them,* E. anophelis* is the most common [5]. The most common clinical manifestations are bloodstream infections without an obvious source and meningitis. They can also cause infections of the lungs, airways, heart (endocarditis), skin and soft tissues, urinary tract, bones, and surgical sites [3]. *Elizabethkingia* spp. are intrinsically multidrug-resistant and can cause nosocomial outbreaks [4]. The first case of human infection with* E. anophelis* was reported in Central Africa in 2013 [6]. Since then, it has emerged as an important human pathogen worldwide. In Japan, only three cases of *E. anophelis* infection have been reported [7,8]. However, there are a few reports of *Elizabethkingia* spp. infection in dialysis patients. We herein report a case of a 68-year-old hemodialysis patient with meningitis due to *E. anophelis* bacteremia. Although the meningitis improved with antimicrobial therapy, the patient developed hemorrhagic shock due to an iliopsoas hematoma along with nonocclusive mesenteric ischemia (NOMI), resulting in death. Data on reports of *E. anophelis* infection in hemodialysis patients are scarce, and its associated meningitis and optimal management strategies in this population remain poorly characterized. Dialysis patients are particularly vulnerable because they frequently experience repeated healthcare exposure through vascular access and potential contact with water-associated healthcare environments. Therefore, given the presence of *E. anophelis* in hospital water systems, including plumbing and drains, as well as its intrinsic multidrug resistance, it should be regarded as a pathogen of particular concern in dialysis patients. We review our case and previous reports of *E. anophelis* infections in hemodialysis patients.

## Case presentation

A 68-year-old man with end-stage renal disease due to diabetic kidney disease, who had received hemodialysis three times per week for four years, was transferred to our hospital because of fever and impaired mobility. The patient’s comorbidities included diabetes mellitus, chronic hepatitis C, chronic obstructive pulmonary disease (COPD), chronic heart failure with pacemaker implantation, and atrial fibrillation. The patient had been taking prednisolone 40 mg/day for five days, starting 11 days before admission for an acute exacerbation of COPD. His clinical findings on admission were as follows: blood pressure, 160/117 mmHg; pulse rate, 108 beats/min with irregular rhythm; body temperature, 39°C; height, 171 cm; and weight, 67.6 kg. Percutaneous oxygen saturation under nasal oxygen support (4 L/min) was 97%. He was alert but disoriented (Glasgow Coma Scale 14; E4, V4, M6) and had bilateral lower-extremity pain that emerged seven days before admission. An examination of his heart, lungs, and abdomen revealed no remarkable findings. A type of vascular access was an arteriovenous graft in the right forearm, and there were no local signs of infection at the access site. Laboratory findings on admission are shown in Table 1.

**Table 1:** Laboratory findings on admission Ab: antibody; Ag: antigen; ALP: alkaline phosphatase; ALT: alanine aminotransferase; APTT: activated partial thromboplastin time; AST: aspartate aminotransferase; BNP: brain natriuretic peptide; BUN: blood urea nitrogen; CK: creatine kinase; Cre: creatinine; CRP: C-reactive protein; γ-GTP: gamma-glutamyl transpeptidase; HBV: hepatitis B virus; HCV: hepatitis C virus; INR: international normalized ratio; LDH: lactate dehydrogenase; PCR: polymerase chain reaction; PT: prothrombin time; PTH: parathyroid hormone; SARS-CoV-2: severe acute respiratory syndrome coronavirus 2; TIBC: total iron binding capacity; UA: uric acid; WBC: white blood cell

Parameter	Value (reference range)
Hematology	
WBC count, /µL	10830 (3300-8600)
neut/lym/mono %	87.6/3.4/8.9
Hemoglobin, g/dL	14.0 (13.7-16.8)
Platelet count, 10^4^/µL	11.7 (15.8-34.8)
PT, %	102 (70-130)
PT-INR	0.99 (1.00)
APTT, seconds	23.6 (24-34)
Fibrinogen, mg/dL	502 (200-400)
Blood chemistry	
Cre, mg/dL	8.12 (0.65-1.0)
BUN, mg/dL	52.6 (8.0-20.0)
Total protein, g/dL	7.2 (6.6-8.1)
Albumin, g/dL	3.7 (4.1-5.1)
UA, mg/dL	6.1 (0.0-7.0)
Na, mEq/L	132 (138-145)
K, mEq/L	5.2 (3.6-4.8)
Cl, mEq/L	92 (101-108)
Ca, mg/dL	8.8 (8.8-10.1)
P, mg/dL	6.9 (2.7-4.6)
Mg, mg/dL	2.3 (1.8-2.3)
AST, U/L	27 (13-30)
ALT, U/L	19 (10-42)
LDH, U/L	279 (124-222)
ALP, U/L	62 (106-322)
γ-GTP, U/L	48 (13-64)
CK, U/L	881 (59-248)
Glucose, mg/dL	247 (73-109)
Glycoalbumin, %	17.2 (11.0-16.0)
CRP, mg/dL	19.93 (0.0-0.14)
Fe, µg/dL	17 (40-188)
TIBC, µg/dL	154 (165-436)
BNP, pg/mL	315 (<18.4)
Immunology	
Ferritin, ng/mL	1074 (21.8-274)
Intact PTH	77 (10-65)
HBV surface Ag	negative
HCV Ab	positive
Influenza A/B-PCR	negative/negative
SARS-CoV-2 PCR	negative

**Figure 1:**
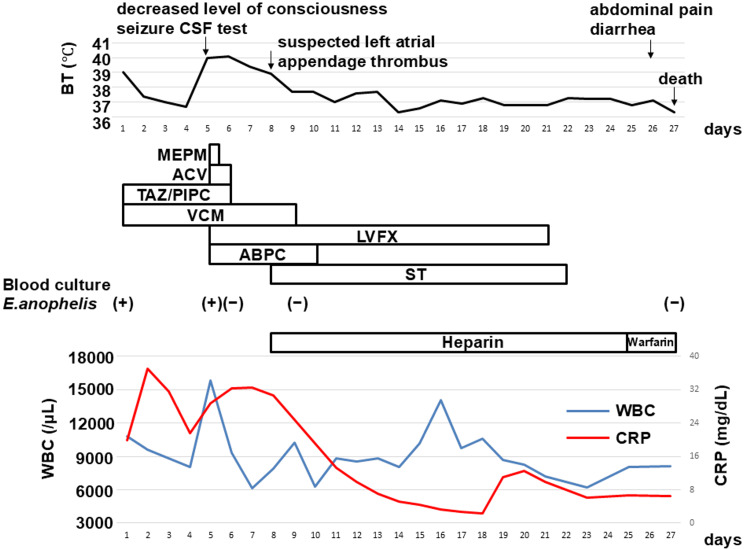
Clinical course of the patient ABPC: ampicillin; ACV: acyclovir; BT: body temperature; CRP: C-reactive protein; CSF: cerebrospinal fluid; LVFX: levofloxacin; MEPM: meropenem; ST: sulfamethoxazole/trimethoprim: TAZ/PIPC: tazobactam/piperacillin; VCM: vancomycin; WBC: white blood cells​​; *E. anophelis: Elizabethkingia anophelis* Despite treatment with tazobactam/piperacillin and vancomycin, fever and inflammatory markers persisted. Blood cultures grew *Elizabethkingia* spp., later identified as *E. anophelis*. On the 5th hospital day, the patient developed seizures and impaired consciousness, and cerebrospinal fluid analysis confirmed bacterial meningitis, prompting a switch to levofloxacin plus sulfamethoxazole-trimethoprim. Transesophageal echocardiography revealed spontaneous echo contrast in the left atrial appendage, and anticoagulation was initiated. Although meningitis improved after two weeks, the patient developed hemorrhagic shock due to retroperitoneal hematoma with severe anemia and died on the 27th hospital day

Blood tests revealed hyperleukocytosis, thrombocytopenia, hypoalbuminemia, electrolyte imbalance, elevated lactate dehydrogenase, elevated glycoalbumin, elevated creatine kinase, elevated brain natriuretic peptide, and elevated C-reactive protein (CRP). Serum ferritin was elevated. The patient was negative for hepatitis B virus, influenza A and B virus, and severe acute respiratory syndrome coronavirus-2 infections. Chest computed tomography (CT) revealed emphysematous changes without definitive radiographic evidence of pneumonia. The clinical course of the patient is shown in Figure 1.

Empirical antimicrobial therapy with tazobactam/piperacillin (TAZ/PIPC) and vancomycin was initiated. Subsequent blood cultures yielded *Elizabethkingia* spp., and because the isolate was susceptible to TAZ/PIPC (Table 2), this regimen was continued. 

**Table 2:** Antimicrobial susceptibility testing of Elizabethkingia anophelis MIC: minimum inhibitory concentration; R: resistant; S: susceptible

Antibiotic	MIC (µg/mL)	Interpretation
Ampicillin	>=32	-
Piperacillin	>=128	R
Amoxicillin/clavulanic acid	16	-
Ampicillin/sulbactam	>=32	-
Tazobactam/piperacillin	<=16	S
Cefaclor	>=32	-
Cefazolin	>=32	-
Cefotiam	>=32	-
Cefditoren	>=4	-
Cefpodoxime	>=8	-
Cefotaxime	>=64	R
Ceftriaxone	>=64	R
Ceftazidime	>=32	R
Cefcapene	>=4	-
Cefozopran	>=32	-
Cefepime	>=32	R
Cefmetazole	<=16	-
Cefoperazone/sulbactam	<=16	-
Latamoxef	>=16	-
Flomoxef	32	-
Aztreonam	>=32	R
Imipenem	>=16	R
Meropenem	>=16	R
Amikacin	>=64	R
Gentamicin	>=16	R
Tobramycin	>=16	R
Minocycline	<=4	S
Levofloxacin	0.5	S
Ciprofloxacin	0.5	S
Fosfomycin	>=32	-
Sulfamethoxazole/trimethoprim	<=2/38	S

Despite the antimicrobial therapy, high fever persisted, and serum levels of CRP failed to improve. On the 5th hospital day, the patient developed a decreased level of consciousness accompanied by a seizure. An examination of cerebrospinal fluid (CSF) showed increased protein levels (260 mg/dL; reference range: 8-43), decreased glucose levels (16 mg/dL; reference range: 50-80), and an elevated cell count (313 µL; reference range: ≦5) with predominance of polymorphonucleocytes (79%), bacterial meningitis was suspected, and combination antimicrobial therapy was initiated. Although *Elizabethkingia* spp. were not isolated from the CSF culture, blood cultures remained positive. In addition, because the FilmArray® meningitis/encephalitis panel showed entirely negative results for other common causative pathogens of meningitis, meningitis due to *Elizabethkingia* spp. was considered, and the antimicrobial regimen was ultimately switched to levofloxacin (LVFX) plus sulfamethoxazole-trimethoprim (ST) according to the susceptibility profile (Table 2) and CSF penetration. Subsequent integrated assessment of 16S rRNA and rpoB gene sequence analyses with phenotypic characterization was performed at the Yokohama City Institute of Public Health, and *Elizabethkingia* spp. isolate was identified as *E. anophelis*. In addition, a transesophageal echocardiography revealed spontaneous echo contrast in the left atrial appendage, and anticoagulation therapy was initiated on the 8th hospital day. The activated partial thromboplastin time was controlled under approximately 50 seconds without marked prolongation, and the international normalized ratio remained below 1.5. After two weeks of the antimicrobial therapy, serum levels of inflammatory markers, the CSF findings (protein levels: 85 mg/dL; glucose levels: 103 mg/dL; cell counts: 7 µL), and the patient’s level of consciousness improved, indicating resolution of the meningitis; however, the patient developed sudden abdominal pain and severe diarrhea on the 26th hospital day. Blood tests revealed severe anemia, with a hemoglobin level of 5.9 g/dL. Contrast-enhanced abdominal CT demonstrated a retroperitoneal hematoma centered on the right iliopsoas muscle, suggesting hemorrhagic shock, and marked colonic gas accumulation and thinning of the intestinal wall were observed. There was no marked over-anticoagulation at the time of bleeding. The patient was deemed unsuitable for surgical intervention and died the following day. An autopsy was performed after obtaining consent from the patient’s family. Pathological findings revealed that the bowel showed marked intraluminal gas accumulation and thinning of the intestinal wall on gross examination. Microscopic evaluation of the colon revealed decreased mucosal epithelial density and mucosal atrophy consistent with ischemic injury; however, there was no evidence of mural necrosis or hemorrhage, indicating NOMI. Notably, there was no intradialytic hypotension or use of vasopressors prior to the onset of NOMI. The iliopsoas hematomas were bilateral, with the right-sided hematoma measuring approximately 20 cm in diameter. No valvular vegetations were identified within the heart. The cause of death was determined to be hemorrhagic shock due to the iliopsoas hematoma, in combination with NOMI.

## Discussion

*Elizabethkingia* spp. comprises three medically important species: *E. anophelis*, *E. meningoseptica*, and *E. miricola*. For *E. meningoseptica*, there have been several reports as a nosocomial pathogen in hemodialysis patients in intensive care units [9], as well as a causative pathogen of peritonitis in peritoneal dialysis patients [10,11]. Likewise, for *E. miricola*, sporadic reports have documented catheter-related infection in a hemodialysis patient [12] and peritonitis in peritoneal dialysis patients [13]. We reviewed PubMed and Google Scholar and found three other cases of *E. anophelis* infection in hemodialysis patients [5,14] (Table 3). In those cases,* E. anophelis* was detected in the blood specimens, presenting as either catheter-related bloodstream infection (CREBSI) or pneumonia, and LVFX was the most frequently used antimicrobial agent; clinical outcome was favorable.

**Table 3:** Clinical characteristics of four cases of Elizabethkingia anophelis infection in hemodialysis patients AVR: atrial valve replacement; CHF: chronic heart failure; COPD: chronic obstructive pulmonary disease; CREBSI: catheter-related bloodstream infection; DM: diabetes mellitus; HT: hypertension; n.d.: not data; RA: rheumatoid arthritis

Case no./ref.	Age/sex	Comorbidities	Community- or hospital-acquired	Positive culture site	Diagnosis	Treatment	Outcome
1/Lau SK, et al. [5]	35/F	Epilepsy, HT	Hospital	Blood	CREBSI	Levofloxacin	Survived
2/Lau SK, et al. [5]	58/M	RA, AVR	Hospital	Blood	CREBSI	Tazobactam/piperacillin, levofloxacin	Survived
3/Güler E, et al [14]	72/F	n.d.	n.d.	Blood	Pneumonia	Levofloxacin	Survived
Present case	68/M	DM, chronic hepatitis C, COPD, CHF	Community	Blood, sputum	Meningitis	Levofloxacin, sulfamethoxazole/trimethoprim	Dead

We present three key clinical messages from this case. First, this case was a hemodialysis patient who developed meningitis that was clinically diagnosed as being caused by *E. anophelis*. Although CSF culture was negative, bacterial meningitis caused by *E. anophelis* was considered highly likely based on the positive blood cultures, compatible CSF findings, and the absence of an alternative pathogen. One possible explanation for the negative CSF culture for *E. anophelis* is that antimicrobial therapy had been initiated before CSF collection, thereby reducing the bacterial load in the CSF to below the detection threshold of culture. Indeed, bacterial meningitis with negative CSF culture following antimicrobial treatment has previously been reported in an infant with *E. anophelis* bacteremia [15]. In the present case, there was no report of similar nosocomial infection at the referring clinic, and no environmental survey on dialysis fluid, reverse osmosis water, or surrounding areas in both our facility and the referring clinic was conducted; therefore, the route of the infection remained unknown. Second, the meningitis improved with LVFX plus ST, but not TAZ/PIPC. *E. anophelis* is resistant to the most commonly used β-lactams, including carbapenems [16]. TAZ/PIPC is a reliable choice for *E. anophelis* bacteremia; however, it poorly penetrates the CSF. When considering effective antimicrobial agents against *E. anophelis* meningitis, fluoroquinolones and ST sufficiently penetrate the CSF [17], and the effectiveness of fluoroquinolones and/or ST has been reported in some cases of meningitis caused by *E. anophelis* in adults [8]. In the present case as well, the antimicrobial therapy was ultimately switched to LVFX plus ST according to the susceptibility profile, resulting in improvement of meningitis (Figure 1). Third, the patient died from hemorrhagic shock due to the iliopsoas hematoma and concomitant NOMI, which was considered not to be directly related to the *E. anophelis* infection. Hemorrhagic shock complicated by NOMI warrants particular caution in anticoagulated hemodialysis patients with severe infection. NOMI is a serious, high-mortality (59%) complication in hemodialysis patients [18]. Key risk factors identified include erythropoietin resistance, diabetes mellitus, longer dialysis duration, and intradialytic hypotension [18]. In this case, there was no intradialytic hypotension or use of vasopressors, and the cause of the development of NOMI was unclear.

In recent years, the number of cases of *E. anophelis* infection has been increasing in Asian countries, including Hong Kong [5], Singapore [19], and Taiwan [20]. There is a potential risk of future outbreaks of *E. anophelis* infection in Japan. *E. anophelis* found in the ubiquitous aquatic environment has also been detected around hospital handwashing areas, suggesting that transmission from healthcare workers could be an important cause of nosocomial infections [21]. Alcohol-based hand rub was stated as an effective method in preventing the transmission of *E. anophelis* [21]. Therefore, it is important for healthcare workers involved in dialysis care to use alcohol-based hand rubs to prevent the spread of multidrug-resistant *E. anophelis* nosocomial infections to immunocompetent dialysis patients.

## Conclusions

We reported the fatal case of clinically diagnosed *E. anophelis* meningitis complicated by NOMI in a hemodialysis patient. We reviewed three other cases of *E. anophelis* infection in hemodialysis patients and found that *E. anophelis* was isolated from blood specimens and has been reported to cause CREBSI or pneumonia. LVFX was the most frequently used antimicrobial agent, and clinical outcomes were favorable. In the present case as well, the antimicrobial therapy was ultimately switched to LVFX plus ST, resulting in improvement of meningitis. Although reports of *E. anophelis* infection in hemodialysis patients are rare, *E. anophelis* is a multidrug-resistant pathogen in hospital aquatic environments, causing severe disease in dialysis patients; therefore, clinicians and dialysis healthcare workers should be aware of *E. anophelis* infection.
